# Intubation of the Larynx

**Published:** 1885-12

**Authors:** F. E. Waxham

**Affiliations:** Professor of Diseases of Children, College of Physicians and Surgeons, Chicago; 3449 Indiana Ave.


SUPPLEMENTAL REPORT OF INTUBATION OF THE LARYNX. *By* F. E. WAHNAM, M. D. *Professor of Diseases of Children, College of Physicians and Surgeons, Chicago.*

In the November number of the JOURNAL AND EXAMINER there appeared a report by me of five cases of true croup treated by intubation, since which time six cases more have come under my care.

CASE VI.—Through the courtesy of Drs. Dahlberg and Appleby I was called November 3d to see a little patient, aged twenty months, suffering from pseudo-membranous laryngitis. The child has been sick for three or four days and now was in imminent danger of suffocation. Tracheotomy had been proposed by the attending physicians but refused by the parents, and intubation was the only resort. Assisted by Drs. Dahlberg, Appleby and Helm the operation was performed quickly, and without the least difficulty. The distressing symptoms were relieved as by magic, and the child at once passed into a quiet, refreshing sleep. The little patient took nourishment well and appeared comfortable. On the fourth day the tube was removed with some difficulty. It was held so closely by the chink of the glottis, that it was grasped with difficulty by the extracting instruments. After three attempts, ether was given the child, when it was immediately removed. The patient did well for two days, when increasing dyspnœa indicated a re-formation of the membrane. On the third day it became necessary to re-introduce the tube to prevent suffocation. Considerable muco-purulent matter and false membrane were expelled as the tube was introduced. Again the violent symptoms were overcome. The child wore the tube for two days, when it was again removed, this time at the first attempt.

The respiration continued a little noisy and slightly embarrassed for two or three days, but it was unnecessary to re-introduce the tube and the child made a good recovery.

This is the youngest child that has yet been saved by intubation.

CASE VII —November 9th I was called to see a little girl between four and five years of age, suffering from profound constitutional diphtheria. The child had been sick for five or six days, when the larynx became involved and it was evident that the child must soon die from suffocation unless relieved. Assisted by Dr. Bosworth and Prof. Nelson, intubation was satisfactorily performed and the suffering at once relieved. The child, however, died forty-eight hours later from the profound diphtheritic infection. The tube in this case did all that was expected, and the child died easily.

CASE VIII.—November 13th I was called by Dr. Kossakowski to tube a little child aged two years and two months, suffering from membranous laryngitis. Assisted by Dr. Kossakowski, while the father held the child in his lap, intubation was quickly performed. As the patient lived a long distance from me, a somewhat larger tube than was appropriate for the age, was introduced in order that it would surely be retained.

On the fourth day it was removed, but as it was impossible for the child to yet carry on respiration the smaller tube was introduced, fearing that possibly ulceration might occur from the pressure of the larger tube. The next day the tube was ejected and I was summoned in great haste. The child could have lived but a few hours longer. The dyspnœa was extreme. The larger tube was re-introduced and again death was averted. On the ninth day the tube was again removed, but it was still impossible for the child to live without it. As the tube was re-introduced large quantities to mucus, mucopurulent matter and some shreds of false membrane were expelled. It is now twelve days since intubation was first performed upon the child. As it is in good condition, eating rye-bread, meat and cookies without difficulty, we certainly predict recovery. The tube will be removed to-morrow, when it is confidently expected that it will no longer be needed.

CASE IX.—November 16th I was again called by Dr. Kossakowski to perform intubation upon a little child of three years. The child was semi-comatose and it seemed hopeless to make the attempt. The child, however, revived after the operation, and the next morning took breakfast with the family at the table. The child died forty-eight hours after the operation, from extension of the membrane into the bronchial tubes.

CASE X.—November 20th I was called by Professors Casselberry and Hatfield to perform intubation upon a child five years old. The patient had been sick eight days, and twice the membrane had re-formed after being ejected. There was evidence of extensive exudation into the bronchial tubes, and it was feared that intubation would give no relief. However, large quantities of mucus, muco-purulent matter and broken down membrane were expelled, when the tube was introduced and the urgent dyspnœa greatly relieved.

The child, however, died about fifty hours after the operation, from membranous bronchitis and inflammation of left lung.

CASE XI.—November 21st I was called to perform intubation upon a little boy five years old, a patient of Dr. Valin. The child had suffered from a severe attack of pharyngeal diphtheria, as had two or three other members of the family. There had been extension of the disease into the larynx. and at this time the child was in the agonies of slow strangulation. All hope had been abandoned, but intubation was given a trial. With the assistance of the father and Dr. Valin the tube was introduced without difficulty, and with the usual result of immediately relieving the violent symptoms. The child is still wearing the tube comfortably. This is the fourth day, and the pulse is now 100, temperature 100, respiration 24, and taking nourishment well. It is very likely that recovery will be the happy result in this case.

Of these eleven cases there have been two complete recoveries, and if cases eight and eleven terminate favorably, as now seems more than likely, we shall have 36 per cent. cured by intubation, a result far better than we get from tracheotomy in Chicago. It must be remembered that seven of these cases with two recoveries were but three years of age or under, a period in which tracheotomy is rarely successful.

November 27, 1885.—Cases eight and eleven are doing well without the tube, and convalescence is assured.

3449 Indiana Ave. F. E. WAXHAM, M. D.

